# Synergistic Interactions among Vacuum, Solar Heating, and UV Irradiation Enhance the Lethality of Interplanetary Space

**DOI:** 10.3390/microorganisms12101976

**Published:** 2024-09-29

**Authors:** Andrew C. Schuerger

**Affiliations:** 1 Department of Plant Pathology, University of Florida, Gainesville, FL 32611, USA; schuerg@ufl.edu; Tel.: +1-(321)-261-3774; 2 Space Life Sciences Laboratory, Department of Plant Pathology, University of Florida, 505 Odyssey Way, Merritt Island, FL 32953, USA

**Keywords:** Europa Clipper, Mars Sample Return mission, planetary protection, Mars astrobiology, spacecraft bioburdens

## Abstract

A Planetary Atmospheric Chamber (PAC) was used to create simulations of interplanetary conditions to test the spore survival of three *Bacillus* spp. exposed to interacting conditions of vacuum (VAC), simulated solar heating (HEAT), and simulated solar ultraviolet irradiation (UV). Synergism was observed among the experimental factors for all three *Bacillus* spp. tested that suggested the increased lethality of HEAT and UV when concomitantly exposed to VAC. The most aggressive biocidal effects were observed for assays with VAC + HEAT + UV conditions run simultaneously over short time-steps. The results were used to predict the accumulation of extremely rapid Sterility Assurance Levels (SALs; def., −12 logs of bioburden reduction) measured in a few minutes to a few hours for external surfaces of interplanetary spacecraft. Furthermore, the results were extrapolated to predict that approx. 1 × 10^4^ SAL exposures might be accumulated for external surfaces on the Europa Clipper spacecraft during a 3.5-year transit time between Venus (0.7 AU) and Mars (1.5 AU) during a series of Venus–Earth–Earth gravity assists (VEEGA trajectory) to Jovian space. The results are applicable to external spacecraft surfaces exposed to direct solar heating and UV irradiation during transits though the inner solar system.

## 1. Introduction

Two flagship missions are under development to explore the surfaces of Mars and Europa and eventually return samples from both planetary bodies. Both missions have the potential to detect extant or extinct life elsewhere in the solar system. The Perseverance rover is the first of three spacecraft within the Mars Sample Return (MSR) mission architecture [1,2,3] and is currently collecting and caching rock, regolith, and aeolian dust samples in the Jezero Crater. Cached samples will be collected by a future Sample Return Lander (SRL) and launched into low Mars orbit (LMO) for pick-up by an Earth Return Orbiter (ERO). Earth-to-Mars transits for all three spacecraft will take between 6 and 8 months, and the first two vehicles will operate on the Martian terrain for many months. In contrast, the Europa Clipper (EC) mission is the first of multiple missions to Jovian space to map, land, and potentially return Europan samples to the Earth [4,5,6]. The EC spacecraft will follow a 6.5-year Earth-to-Jupiter cruise phase that will include one flyby of Venus and two flybys of the Earth for gravity assists (i.e., called the VEEGA trajectory). 

Both missions will be exposed to five dominant biocidal space conditions during transit including high vacuum, extreme desiccation, solar heating, solar UV irradiation on external surfaces, and ionizing radiation [7]. The MSR Perseverance rover and the SRL spacecraft will be further exposed to up to 20 biocidal conditions present on the Martian surface including solar UV irradiation, desiccation, volatile oxidants, low pressure, high salt concentrations, extremes in dust pH, an anoxic CO_2_-dominated atmosphere, and periodic solar particle events (to mention a few) [8,9,10,11]. The Mars landers during transit will be encased within cruise-phase systems with heat shields and back shells protecting most surfaces from direct solar heating and solar UV and thus partially shield the spacecraft from the space biocidal conditions listed above. In contrast, the EC and ERO spacecraft will be partially or fully deployed during their interplanetary cruise phases and will receive significantly greater amounts of solar heating and solar UV irradiation during transit. 

In order to protect the scientific integrity of both mission architectures, planetary protection protocols (PPs) have been (Perseverance and EC spacecraft; e.g., [12,13,14,15]), and will be, developed to (i) minimize pre-launch bioburdens on critical subsystems that will directly handle samples; (ii) sterilize and place, behind biobarriers, criticality-1 subsystems that must be free of terrestrial contamination prior to deployment on surface terrains; (iii) streamline collection and caching activities to prevent the recontamination of pre-sterilized components; (iv) select unique materials for specific surfaces to compliment the mitigation of recontamination; and (v) develop quantitative models to predict the inactivation of adhered bioburdens during interplanetary flight. Regarding the latter PP component, three quantitative models for microbial survival in interplanetary space have been developed [16,17,18]. The study by Dillon et al. [16] focused on thermal issues related to landed spacecraft on the Moon. The Lunar Microbial Survival (LMS) model [17] included the interactive effects of solar heating, solar UVC irradiation, vacuum, and ionizing radiation on microbial survival on the Moon. And the Cruise-Phase Microbial Survival (CPMS) model [18] extended the LMS model to predict bioburden inactivation rates for interplanetary spacecraft traversing out to >10 astronomical units (AU). The CPMS model predicts that synergism between solar heating and vacuum for internal surfaces will dominate as biocidal factors out to approx. 10 AU, after which vacuum alone will dominate because of the extremely low cryogenic temperatures beyond the orbit of Saturn [18]. Solar UV irradiation contributes to the sterilization of external spacecraft surfaces out to 100 AU and beyond [18].

The primary objectives of the current research were to (i) characterize the biocidal effects of three interactive factors under simulated space conditions applied concurrently to spores of three *Bacillus* spp.; (ii) determine whether the concomitant effects of vacuum (VAC), simulated solar heating (HEAT), and simulated solar ultraviolet irradiation (UV) exhibited additive or synergistic relationships for killing *Bacillus* spp. spores; and (iii) develop datasets that can be used to validate the LMS and CPMS models and initiate the development of a Mars Microbial Survival (MMS) model. The results are expected to directly impact predictions on how microbial bioburdens can be inactivated on multiple spacecraft within the MSR and EC mission architectures. 

Three *Bacillus* spp. were selected for this research because their dormant spores are highly resistant to the biocidal conditions in interplanetary space [7,8,15] and they are used as proxies for monitoring the total bioburdens on spacecraft surfaces [19]. *Bacillus subtilis* was selected because it is a lab standard for planetary protection research, *B. pumilus* was selected as a UV-resistant species, and *B. atrophaeus* was selected as a heat-tolerant species [19].

## 2. Material and Methods

### 2.1. Microbial Protocols

Three *Bacillus* spp. were used to characterize the biocidal effects of VAC, HEAT, and solar UV irradiation. *Bacillus atrophaeus* ATCC 9372 spores were purchased commercially from Mesa Labs (Bozeman, MT, USA). *Bacillus pumilus* SAFR-032 and *B. subtilis* 168 spores were produced in-house according to the procedures of Mancinelli and Klovstad [20] and Schuerger et al. [21]. Spores were concentrated in sterile deionized water (SDIW) and maintained at 4 °C until required. 

Spore suspensions were calibrated to yield 2 × 10^7^ spores/mL with a UV-VIS spectrometer set at an optical density (OD) of 600 nm. Spores were applied to uncoated aluminum 6061 coupons (quote 27118101, Seton, Inc., Williamsville, NY, USA) at the rate of 100 µL per coupon (i.e., 2 × 10^6^ spores/coupon), stored at 24 °C overnight (18–20 h), and dried under a flow of filtered–sterilized air in a NuAire biosafety hood (NU-440-600, Class 2, Type A2, Plymouth, MN, USA) according to protocols described by Schuerger [22] and Schuerger and Headrick [23]. Individual coupons with dried spores were selected based on uniformity of the spore monolayers and the diameters of the monolayers being approx. 1.3 cm. After exposing spore monolayers to individual treatments (see below), the spores were removed from coupons by a Most Probable Number (MPN) silica-sand-vortexing extraction protocol that has an extraction efficiency of 95–99% from aluminum coupons [22]. 

### 2.2. Planetary Atmospheric Chamber 

**Figure 1:**
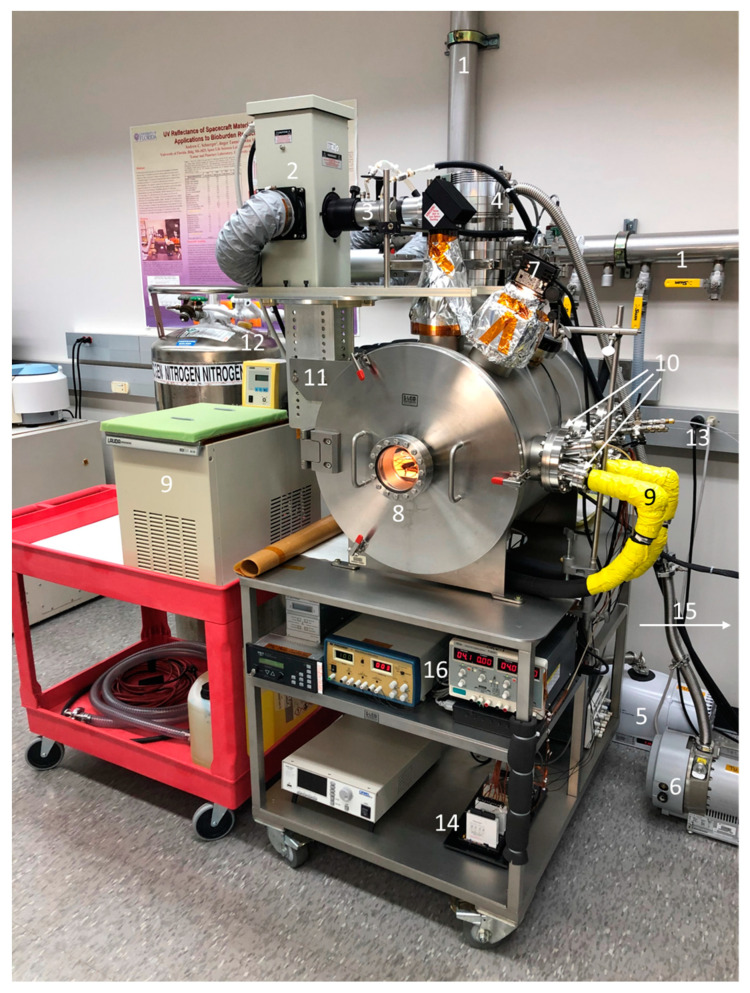
Planetary Atmospheric Chamber (PAC). (1) Vent lines for PAC. (2) UV-VIS illuminator. (3) Six cm water filter to remove thermal IR irradiation. (4) Agilent Turbo pump. (5) Roughing scroll pump for Mars surface conditions. (6) Roughing scroll pump for Agilent Turbo pump. (7) Tungsten-halogen lamp. (8) Front port composed of non-UV-transmitting borosilicate glass. (9) Lauda water chiller of the cryogenic cooling system for temperature control down to −10 °C (with yellow pipes). (10) Multi-port 6” CF flange with three 2.5” CF flanges. (11) Steel support structure for the UV-VIS illuminator. (12) Liquid nitrogen dewar for temperature control down to −100 °C. (13) All electrical power was located on the back wall. (14) Opto-22 data logging system. (15) Tanks of Mars gas located to the right of the PAC. (16) DC power supplies for internal PAC sensors.

Simulated interplanetary conditions were created within a Planetary Atmospheric Chamber (PAC; Figure 1) originally described by Schuerger et al. [24,25] but upgraded since then. The PAC system was recently fitted with a new cryogenic liquid cooling system that was able to hold temperatures between −10 and 100 °C. The original PAC design relied on a liquid nitrogen (LN2) cold plate (model TP-1265 Thermal Platform, Sigma Systems Corp., San Diego, CA, USA). The newer cryogenic thermal plate was located on the upper surface of the LN2 system and used a polypropylene antifreeze fluid in a Lauda recirculating bath to control temperature (Figure 1, yellow lines). Using both systems in a coordinated manner, the LN2 system could rapidly cool or warm the samples to the desired setpoints within 20 min, and the cryogenic system would be used to hold those setpoints over longer periods of time. 

The interplanetary conditions created within the PAC are described below for specific experiments. However, VAC was controlled at 0.05 (5 × 10^−2^) mbar for Exp-1 and 5 × 10^−6^ mbar for Exps-2, -3, and -4. The lower pressures for Exp-2, -3, and -4 were possible after the addition of an Agilent Turbo Pump system (Figure 1; TwisTorr 304 FS turbo pump and controller, Agilent Technologies, Inc., Wilmington, DE, USA) to the PAC after Exp-1 was completed. 

*Experiment 1.* Doped coupons with 2 × 10^6^ spores per coupon for each *Bacillus* spp. were placed within sterile glass petri dishes on the cryogenic cold-plate surface. The PAC system was sealed and depressurized to 3 × 10^−2^ mbar [VAC] (i.e., via a Variant IDP-7 scroll pump used as the vacuum controller; Figure 1; see Schuerger et al. [24,25]). High temperature was set at 100 °C [HEAT] and held for 12, 24, or 48 h at low pressure. The PAC was then cooled to lab conditions between 22 and 24 °C and vented with filter-sterilized ultra-high-purity nitrogen (UHP N_2_) gas. The treated coupons were then processed by the MPN-sand-vortexing protocol. Non-heated controls were exposed to 25 °C within the PAC system set at either 1013 or 10^−2^ mbar. 

*Experiment 2 (low UVC).* The UV illumination system on the PAC was calibrated to deliver a UVC flux of 0.672 W m^−2^ at the upper surfaces of the aluminum coupons. The UVC flux was selected to simulate approx. 3.16 AU, which occurs within the asteroid belt circumnavigating the Sun [18]. The temperature was set at 25 °C for Exp-2.

Aluminum coupons were created as described for Exp-1, placed in sterile glass petri dishes, had their glass tops removed, and had the PAC closed and depressurized to 0.1 mbar. After the pressure reached 0.1 mbar, the Turbo pump was engaged, and the PAC internal pressure dropped to ~5 × 10^−5^ mbar within 2 h. The high VAC conditions reached the lowest possible level within the PAC and Turbo pumping system of 5 × 10^−6^ mbar after ~16–18 h (i.e., in general, the PAC was equilibrated to 5 × 10^−6^ mbar overnight before UV exposures). After 24 h, the UV light beam was allowed to enter the PAC system through a fused-silica glass port striking the exposed spore monolayers in the glass petri dishes. The UV exposures were conducted for 0, 1, 5, 10, or 30 min. After the UV exposures were completed, the PAC chamber was maintained at the setpoints until it was slowly vented at 48 h with filter-sterilized UHP N_2_ gas, and the coupons were processed for survivors with MPN assays. Total elapsed time for samples at 5 × 10^−6^ mbar was 48 h.

*Experiment 3 (high UVC).* In a separate series of assays, the PAC UV illuminator was recalibrated to deliver a higher dosage of UVC irradiation (3.6 W m^−2^) to the upper surfaces of the spore monolayers. The higher UVC flux was similar to the UVC flux at 1.36 AU, close to perihelion for low Mars orbit (LMO) [18]. Experiment 3 was identical to Exp-2 except for the difference in the delivered UVC flux to the spore monolayers. Total elapsed time for samples at 5 × 10^−6^ mbar was 48 h. The temperature was set at 25 °C for Exp-3.

**Figure 2:**
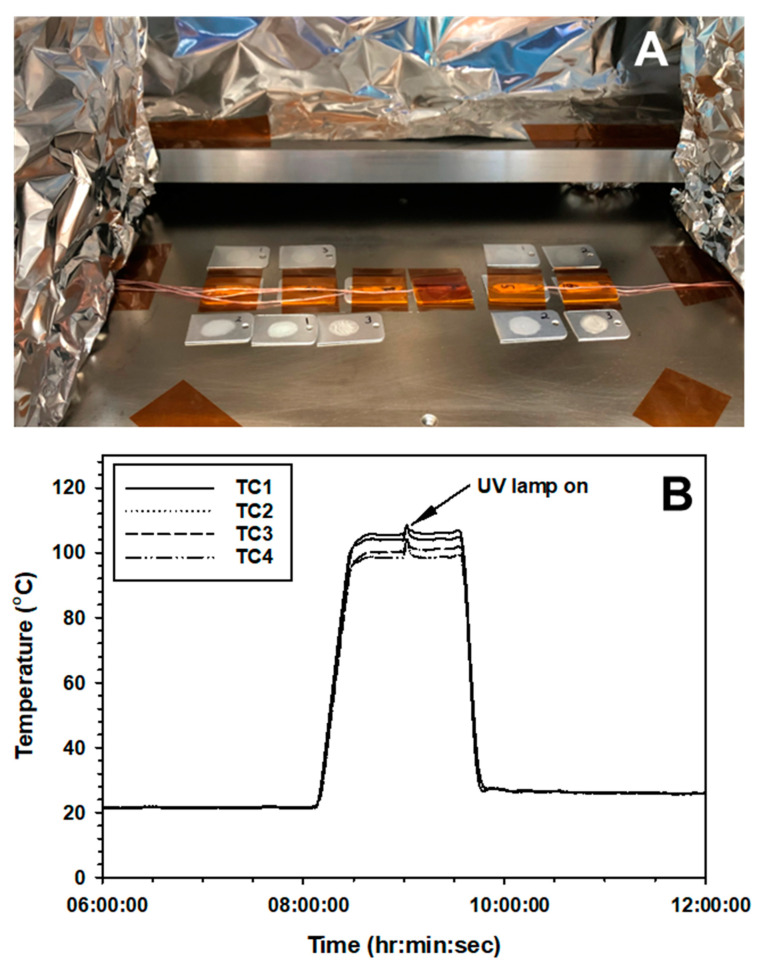
Setup within the PAC system for Experiment 4. (**A**) The LN2 surface was sterilized with 70% isopropanol prior to placing doped coupons of the three *Bacillus* spp. directly on to the LN2 thermal control plate. A row of thermocouples was taped down to the LN2 surface with 4-mil Kapton tape and were aligned with two rows of aluminum coupons. (**B**) Temperature profile of one VAC + HEAT + UV assay. The ramp-up to 100 °C required approx. 20 min and exhibited an approx. 10 min shoulder between 90 and 100 °C. The separate thermocouples exhibited a slight divergence due to the placement on the LN2 surface. The small spike (arrow) of all TCs at approx. 0900 during this assay indicated the time when the UV irradiation source was turned on.

*Experiment 4.* The final series of assays were designed to characterize the synergistic biocidal effects of interactions among high vacuum (VAC), high temperature (100 °C; HEAT), and a simulated solar UVC flux at approx. 1.36 AU (3.6 W m^−2^; UV). To enhance thermal control during Exp-3, doped coupons of all three *Bacillus* spp. were randomly distributed along a transect on top of the LN2 thermal control plate in the PAC (Figure 2A). The LN2 surface was surface sterilized with 70% isopropanol prior to placing the coupons directly on the cold-plate surface. Non-doped and sterile coupons were tested in the protocol and no surface contamination was detected. 

Spores of all three *Bacillus* spp. were exposed together to one of five treatments in a completely randomized design. The five treatments were as follows: (1) lab controls held at 25 °C and 1013 mbar for 48 h, (2) VAC only at 5 × 10^−6^ mbar, (3) VAC + HEAT exposures, (4) VAC + UV exposures, and (5) VAC + HEAT + UV exposures. 

All coupons were exposed to high vacuum for 48 h. After 24 h (i.e., in the middle of the VAC phase), the LN2 and cryogenic thermal systems were coordinated to rapidly raise the temperature of the coupons to 103 °C (+/−5 °C) for 1.25 h with warming and cool-down timesteps of 30 min each (Figure 2B; see Table S1). In the middle of the HEAT cycle, the UV irradiation was allowed to enter the PAC system for precisely 2 min. Time-steps for each factor were selected as ‘*sublethal*’ doses of the parameters. Longer heating or UV exposures would have resulted in fully inactivating (i.e., killing) the spores applied to the aluminum coupons. The goal here was to investigate synergistic interactions among sublethal dosages of three space biocidal parameters. 

### 2.3. Statistical Analyses 

The PC-based software Statistical Analysis System (SAS) version 9.4 (SAS Institute, Inc., Cary, NC, USA) was used for all analyses. All raw data for Exps-1 through -4 are given in Tables S2–S5, respectively. Survival data from the MPN assays for all coupons were transformed to log10(N/N_0_) values in which N equaled the surviving numbers of spores after exposures and N_0_ equaled T = 0 survivors from coupons prior to exposure. If no surviving spores were detected for a given coupon (called ‘*non-detects*’; i.e., below the minimum detection limit of 10 viable spores per coupon), a random number between 0 and 1 was generated with a hand calculator (HP 11C, Hewlett-Packard, Palo Alto, CA, USA) and assigned to that cell in the Excel spreadsheet because ANOVA does not recognize zeroes when log-transformations are employed. 

When all replicates in a given sample treatment were *non-detects*, those treatments were deleted from the analyses. Retaining treatments in the analyses and plots when all replicates are *non-detects* artificially flattens the linear models, and thus, obscures the synergism of the parameters (divergent slopes) at shorter time-steps. 

**Figure 3:**
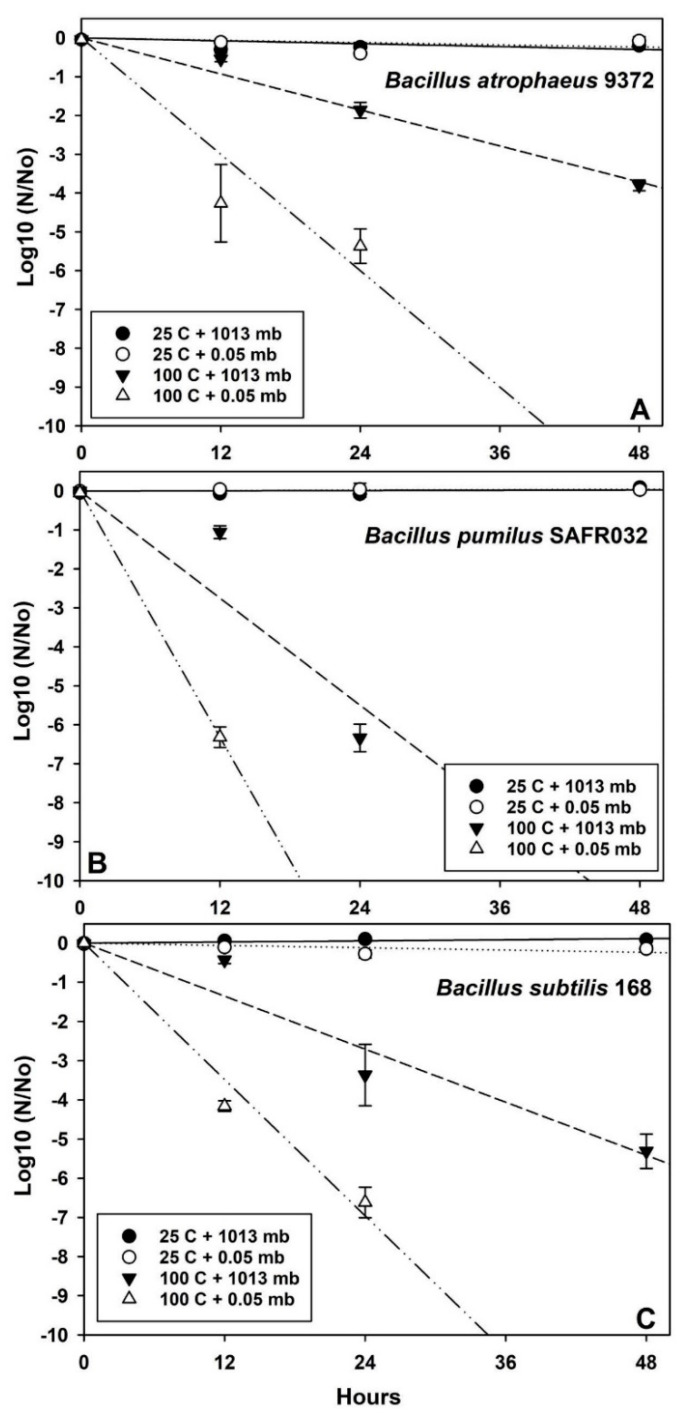
Linear plots for interactive effects of vacuum (VAC) and heat (HEAT) on three *Bacillus* spp. Treatments that had no detected survivors at 24 h (*B. pumilus*) or 48 h (all three *Bacillus* spp.) when exposed to 100 °C at 0.5 mbar were deleted from the analyses (see supplementary data tables for all experimentally derived data). Synergistic interactions were observed between VAC and HEAT for *B. atrophaeus* ATCC 9372 (**A**), *B. pumilus* SAFR-032 (**B**), and *B. subtilis* 168 (**C**) (i.e., divergent slopes; *p* ≤ 0.05; n = 6). Interactions of VAC + HEAT appeared to cause the most biocidal effect on *B. pumilus* spores (i.e., greater negative slopes).

**Figure 4:**
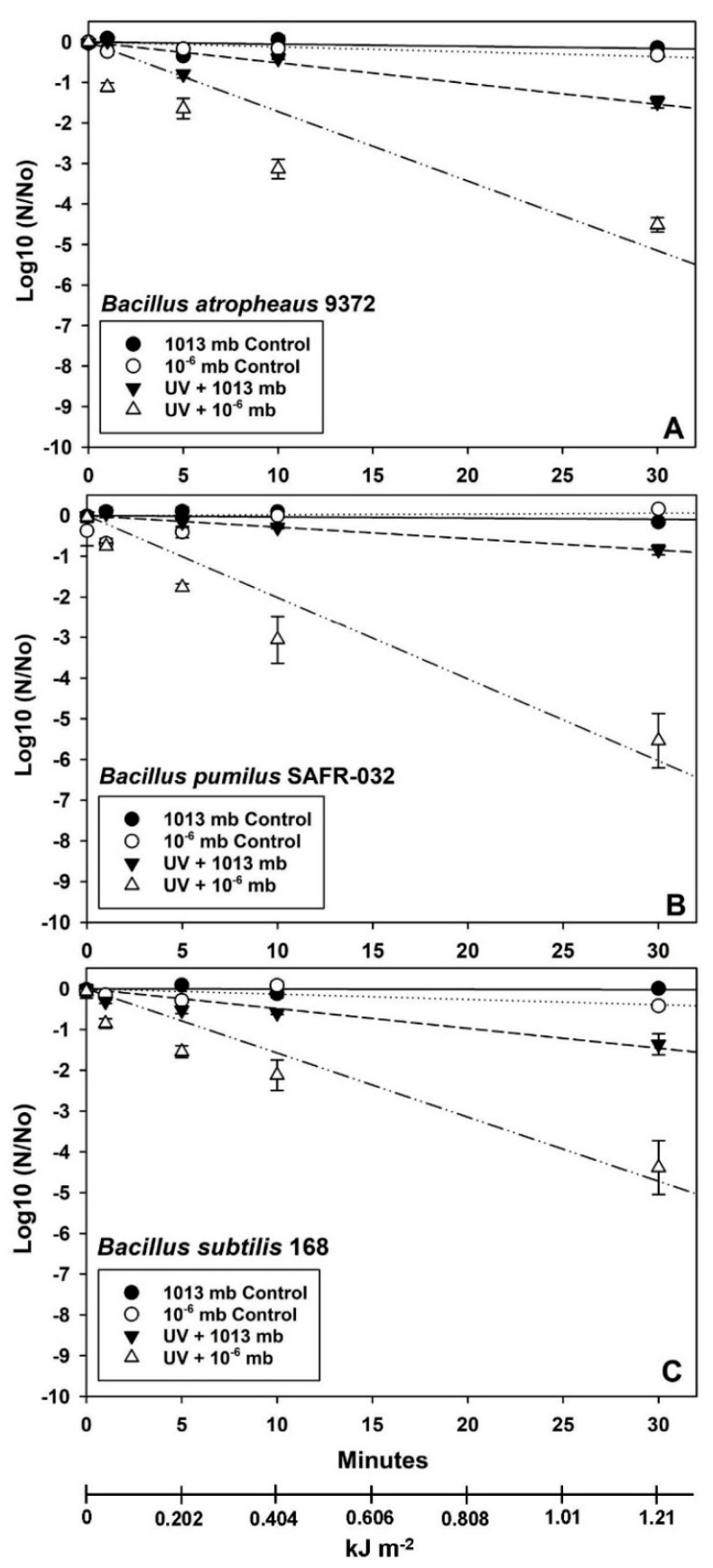
Linear models for interactive effects of vacuum (VAC) and low UV flux (0.672 W m^−2^) on three *Bacillus* spp. Synergistic interactions were observed between VAC and low UV for *B. atrophaeus* ATCC 9372 (**A**), *B. pumilus* SAFR-032 (**B**), and *B. subtilis* 168 (**C**) (i.e., divergent slopes; *p* ≤ 0.05; n = 4). Interactions of VAC + low UV appeared similar among the three *Bacillus* spp. tested. The steepest slopes for the VAC + low UV treatments were observed for (in priority order) *B. pumilus*, *B. atrophaeus*, and *B. subtilis* (see Table S6 for slope values), indicating that the highest biocidal effect was observed for *B. atrophaeus* spores. Independent treatment variables are presented in minutes and kJ m^−2^.

**Figure 5:**
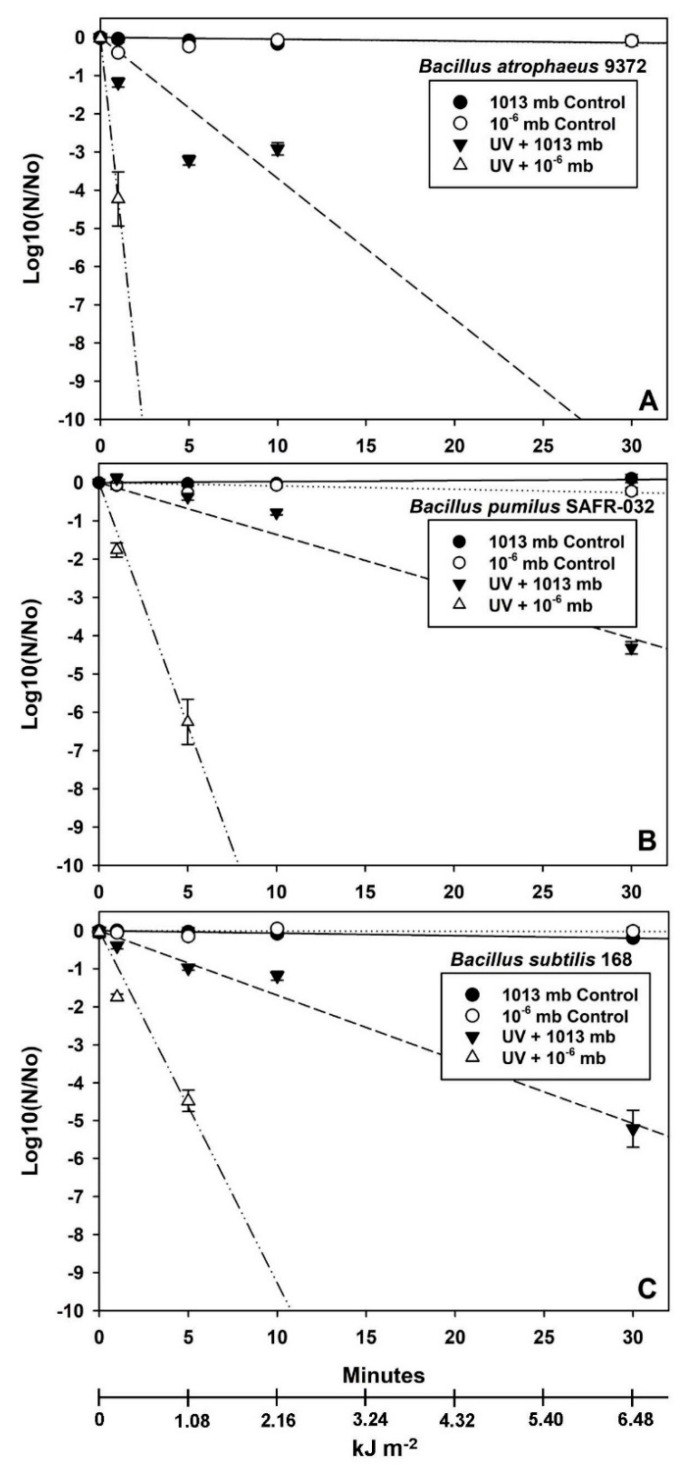
Linear models for interactive effects of vacuum (VAC) and high UV flux (3.6 W m^−2^) on three *Bacillus* spp. Only the linear 1st-phase models (see text) for VAC + high UV are plotted here for each species. Synergistic interactions were observed between VAC and high UV for *B. atrophaeus* ATCC 9372 (**A**), *B. pumilus* SAFR-032 (**B**), and *B. subtilis* 168 (**C**) (i.e., divergent slopes; *p* ≤ 0.05; n = 4). The steepest slopes for the VAC + high UV treatments were observed for (in priority order) *B. atrophaeus*, *B. pumilus*, and *B. subtilis* (see Table S6 for slope values), indicating that the highest biocidal effect was observed for *B. atrophaeus* spores. Independent treatment variables are presented in minutes and kJ m^−2^.

**Figure 6:**
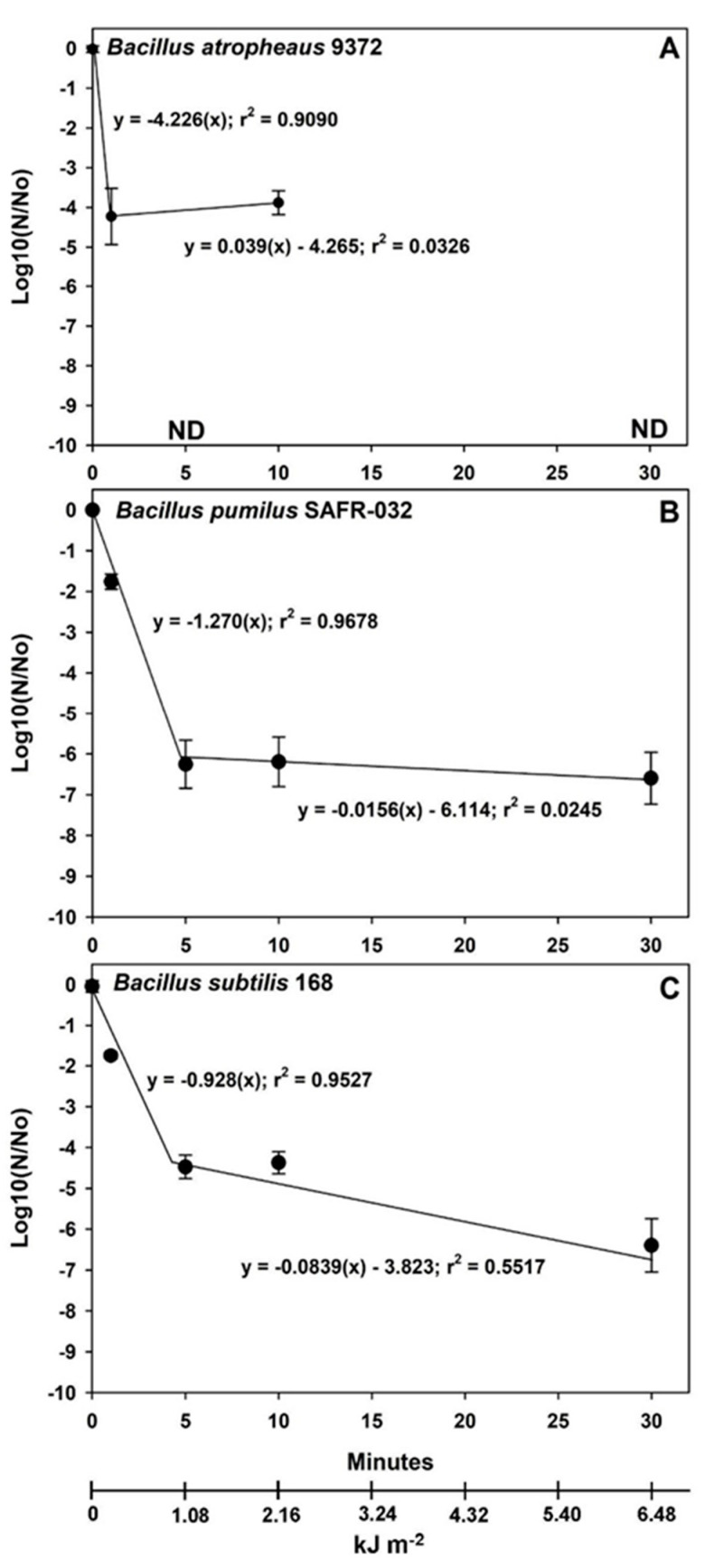
Biphasic linear models for interactive effects of vacuum (VAC) and high UV flux (3.6 W m^−2^) on three *Bacillus* spp. Linear models for the 1st phases (i.e., steep slopes) are interpreted to represent the inherent biocidal effects of UV irradiation on *B. atrophaeus* ATCC 9372 (**A**), *B. pumilus* SAFR-032 (**B**), and *B. subtilis* 168 (**C**) spores (*p* ≤ 0.05; n = 4). The 2nd phases (i.e., shallow slopes) are interpreted to represent low numbers of outliers in which spores are partially shielded by surface defects or multi-layered stacking of spores on the aluminum coupons. Independent treatment variables are presented in minutes and kJ m^−2^. Abbreviations: ND = no detected spores on all replicates.

**Figure 7:**
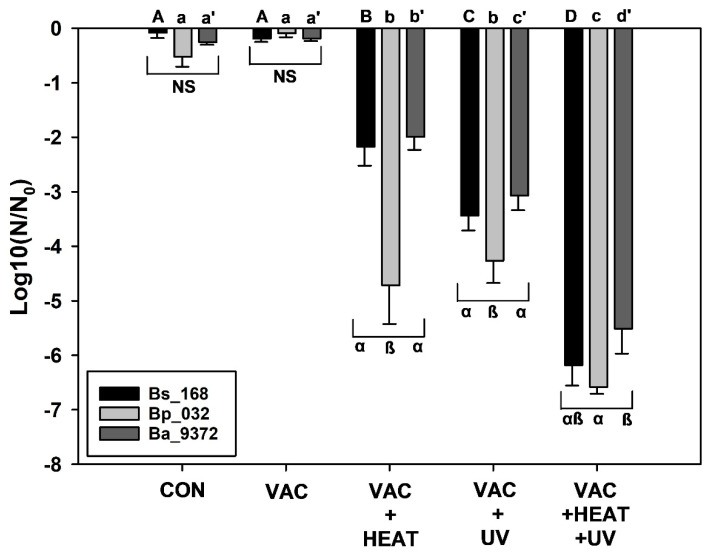
Interactive effects for spores exposed to vacuum (VAC), 100 °C (HEAT), and high UV flux (UV) for *Bacillus atrophaeus* 9372, *B. pumilus* SAFR-032, and *B. subtilis* 168. Data for individual *Bacillus* spp. were analyzed separately. Data were log-transformed prior to ANOVA and protected least-squares mean (LSM) separation tests (*p* ≤ 0.05; n = 9). Capital letters along the top of the plot are LSM tests for *B. subtilis*; small letters are for LSM tests for *B. pumilus*; and prime letters are for LSM tests for *B. atrophaeus*. LSM comparisons were also examined for clusters of the three *Bacillus* spp. for each treatment combination (i.e., brackets and Greek letters within the plot).

Data in Figure 3, Figure 4 and Figure 5 were analyzed with ANOVA using *PROC GLM* statements (*p* ≤ 0.05; n = 4 or 6; see Supplementary Tables S1–S6). Slopes and *p*-values for linear models were compared with *ESTIMATE* statements within individual *Bacillus* spp. for each experiment. Data in Figure 6 were analyzed with *PROC REG* for linear models in which the 1st and 2nd phases of the biphasic responses were analyzed separately. Spline points for the intersections of the two phases in Figure 6 were estimated with the online app www.WolframAlpha.com (last accessed on 7 August 2024) in ‘approximate mode’ [e.g., use the phrase ‘solve y_1_ = mx + b (1st phase) and y_2_ = mx + b (2nd phase)’]. Data for Exp-4 (Figure 7) were analyzed with ANOVA followed by protected least-squares mean separation (LSM) tests (*p* ≤ 0.05; n = 9) run for individual *Bacillus* spp. (i.e., LSM comparisons were limited to each bacterium tested separately). In Figure 3, Figure 4 and Figure 5, all datasets were normalized to zero for log10(N/N_0_) transformations yielding y-intercepts that were identical, and thus, slopes of different linear models could be compared directly to determine divergence from equality. Slopes for the 1st-phase linear models for Figure 3, Figure 4 and Figure 5 are given in Table S6. If the slopes of separate linear models diverged, synergism of the biocidal factors was likely. All bars in the figures are standard errors of the means. 

## 3. Results 

Four experiments were conducted to characterize the synergistic interactions among three space biocidal factors (i.e., VAC, HEAT, and UV) encountered during the interplanetary travel of spacecraft. The research was based on several key microbial assumptions for interplanetary spacecraft, including the following: (i) spores on spacecraft surfaces occurred as individual propagules adhered to surfaces as monolayers [13,23]; (ii) the potential effects of multi-layered aggregates [23] of spores were ignored (iii) because *Bacillus* spp. endospores are significantly more resistant to space biocidal conditions than non-spore-forming species [7], the sterilization of vegetative cells was assumed to occur at faster rates for non-spore-forming species, and (iv) spores on external spacecraft surfaces are not shielded from UV exposures during interplanetary travel.

The results across all four experiments were similar. First, two controls were included in each experiment and were composed of (i) a lab control maintained at 1013 mbar (i.e., average sea level pressure) and room temperature (between 22–25 °C) and (ii) a low-pressure control within the PAC that was maintained at 10^−2^ mbar (Exp-1) or 10^−6^ mbar (Exp-2, -3, and -4) and room temperature. In all four experiments, these two controls exhibited similar and not significantly different survival plots (Figure 3, Figure 4 and Figure 5 and Figure 7). The results indicated that there was no apparent biocidal effect of VAC alone on spores of all three *Bacillus* spp. (*p* > 0.05). Second, the biocidal effects of HEAT or UV irradiation were significantly enhanced when VAC was included in the experimental conditions. 

Based on comparing the slopes of the inactivation plots for the HEAT-only assays in Exp-1, *B. atrophaeus* 9372 was the most resistant to high temperature alone (Figure 3A) followed by *B. subtilis* 168 (Figure 3C) and *B. pumilus* SAFR-032 (Figure 3B). In contrast, when considering the synergistic effects of adding high vacuum to the assays, the order of resistance to VAC + HEAT was observed as *B. atrophaeus* ≈ *B. subtilis* > *B. pumilus*.

Vacuum-plus-UV-irradiation assays were split into two different experiments in which a low UVC flux of 0.672 W m^−2^ was tested in Exp-2 (Figure 4; similar to 3.16 AUs in the asteroid belt) and a high UVC flux of 3.6 W m^−2^ was tested in Exp-3 (Figure 5 and Figure 6; similar to Mars perihelion in LMO). The two UVC fluence rates were tested because previous work [26] had established extremely rapid inactivation rates for seven *Bacillus* spp. when exposed to LMO-like UVC rates, and thus, synergism between VAC and UV would have been much more difficult to examine if only the LMO rate of 3.6 W m^−2^ was used. Furthermore, the low UV flux would yield data applicable to interplanetary spacecraft traveling through the asteroid belt [18].

Results for the VAC + low UVC flux demonstrated only moderate inactivation kinetics for UVC exposures when conducted at lab-normal pressures of 1013 mbar for all three *Bacillus* spp. tested (Figure 4). In contrast, when vacuum levels close to 10^−6^ mbar were concurrently used in the VAC + low UVC assays, synergistic interactions were observed that increased the lethalities at 30 min of UV exposure to −5.5 logs for *B. pumilus* and −4.5 logs for *B. subtilis* and *B. atrophaeus*. Thus, the order of resistance to VAC + low UVC was observed to be *B. atrophaeus* ≈ *B. subtilis* > *B. pumilus*. 

In contrast, the VAC + high UVC assays exhibited significantly greater losses of spore viability in which the biocidal effects of adding VAC to the high UV flux were similar among the three *Bacillus* spp. (Figure 5). The order of resistance to VAC + high UVC appeared to be *B. subtilis* > *B. pumilus* > *B. atrophaeus.* Furthermore, the interactive effects of VAC + high UVC generally yielded losses of viability between −4 to −6.5 logs for all three *Bacillus* spp. after only 5 min of LMO simulations. 

All data for Exp-1 (Figure 3) and Exp-2 (Figure 4) were best represented by linear models over the plotted ranges of the independent variables. In Exp-3 (Figure 5), linear models best represented the (i) lab controls, (ii) internal PAC vacuum controls, and (iii) high UVC irradiation at lab atmospheric pressures of 1013 mbar. In contrast, the bioburden reductions for the VAC + high UV exposures followed biphasic responses in which distinct inflection points (i.e., called a spline point) were observed between the 1st phases (i.e., steep slopes) between 0 and 5 min and the 2nd phases (i.e., shallower slopes) between 5 and 30 min (Figure 6). In general, this ‘*tailing effect*’ might be represented by logarithmic decay plots that smoothly transition through the inflection points given in Figure 6. However, a single logarithmic decay plot implies that a single biocidal factor is acting upon survival through the transition from an initial rapid inactivation rate to a more refractory inactivation process. 

It is proposed here that logarithmic decay plots do not accurately represent what is occurring biologically to dried spores on simulated spacecraft materials. It is proposed that the 1st phase represents the inherent biocidal effect of the stressor(s) on spore survival (i.e., VAC + high UVC in this case) and the 2nd phase represents shadowing effects by *Bacillus* spores caught in surface defects or stacked into multi-layers on the aluminum coupons (see [22,23,26]). Figure 6 gives the biphasic linear plots for the VAC + high UVC irradiation data in Exp-3. In all three *Bacillus* spp., the initial 1st phase plots exhibited extremely steep slopes indicating that the inherent biocidal sensitivity of the spores to the interplanetary simulation would follow fast inactivation rates for sun-exposed spacecraft surfaces. The 2nd phases captured shadowing ‘*outliers*’ that did not necessarily represent sensitivity to UVC irradiation. 

Lastly, a three-factor experiment was conducted that combined sublethal doses of VAC + HEAT + high UVC irradiation to examine the interactive effects of these space biocidal conditions on spores of three *Bacillus* spp. Based on the results in Exp-1, -2, and -3, sublethal doses of each parameter were chosen. The VAC exposure was for 48 h in the PAC system for all assays because VAC alone did not appear to induce any discernable biocidal effect in earlier experiments. The HEAT treatment was for only 1.25 h (with short 30 min ramps for heating and cooling; see Figure 2B). And the high UVC irradiation exposure was for only 2 min. These short exposures for HEAT and high UVC should have yielded inactivation rates of less-than −1 log for HEAT for all three *Bacillus* spp. (based on Figure 3) and approx. −2 to −4 logs for high UVC for all three species (based on Figure 5). However, the combinations of the three sublethal doses of the simulated space factors yielded approx. −6.5 to −7 logs of biocidal activity for all three *Bacillus* spp. (Figure 7). 

A Sterility Assurance Level (SAL) can be defined as a bioburden reduction of greater than −12 logs achieved by determining the biocidal effect of a sterilizing protocol on a viable population of 1 × 10^6^ spores (i.e., −6 logs) and extrapolating the inactivation rate to −12 logs [19,27,28]. SAL predictions (Table 1) for the plots in Figure 3, Figure 4 and Figure 5 were generated by dividing −12 logs (i.e., the desired bioburden reduction; 1 SAL) by the negative slope values given in Table S6. SAL values were only generated for the combinations of synergistic factors in Experiments 1, 2, and 3. However, the approach can be used for all linear models with negative slopes.

SAL predictions to achieve bioburden reductions of −12 logs exhibited very short time-steps for all three *Bacillus* spp. against exposures to VAC + HEAT, VAC + low UV, and VAC + high UV (Table 1). In Exp-1, which was on the interactive effects of VAC + HEAT (Figure 3), *B. pumilus* exhibited the shortest SAL time-step of 22.8 h to reach −12 logs. In the VAC + low UV experiment (Exp-2; Figure 4), the SAL predictions for all three *Bacillus* spp. were similar and in the range of 59.7 to 76.3 min. For Exp-3 (Figure 5) (i.e., VAC + high UV), the 1 SAL predictions were extremely fast and on the order of 2.84 min (*B. atrophaeus*), 9.45 (*B. pumilus*), and 12.94 min (*B. subtilis*). 

SAL predictions for the synergistic interactions among the three space biocidal conditions of VAC + HEAT + UV are a bit more cumbersome to derive because the results are not amenable to generating linear models. However, as a first-order prediction, if most or all of the replicates exhibited non-detects in the VAC + HEAT + UV assays (see Table S5), doubling the time-steps for each factor might allow a SAL prediction. Recall that for Exp-4, the VAC was held at 10^−6^ mbar for 48 h, the HEAT was raised to 100 °C for 1.25 h, and the high UVC flux of 3.6 W m^−2^ was applied for 2 min. A 1 SAL prediction of these three factors might be the equivalent of doubling the times for HEAT (e.g., 2.5 h) and high UVC (e.g., 4 min). VAC-only effects are ignored here because the results from all four experiments support the conclusion that VAC had little to no effect when tested alone. Thus, external spacecraft surfaces within the inner solar system would achieve hundreds to thousands of SAL exposures within a few weeks to a few months in space. 

## 4. Discussion

Synergistic interactions among multiple space biocidal conditions provide more accurate predictions on the lethality of the interplanetary environment than single-factor experiments. However, multi-factorial assays are often difficult to complete due to rapidly increasing numbers of combinations of the biocidal factors. For example, if three factors are considered for an assay, the total number of possible combinations are eight (i.e., 2^N^ = 2^3^ = 8 combinations). Four factors quickly escalate to 16 combinations (i.e., 2^4^ = 16). Furthermore, selecting the dominant factors to test often leads to significant knowledge gaps for more subtle interactions. In the current study, the interactive effects of vacuum (VAC), simulated solar heating (HEAT), and simulated solar UV irradiation (UV) were examined in various combinations to examine the potential lethality of interplanetary space on three spore-forming *Bacillus* spp. commonly recovered from spacecraft surfaces. 

Vacuum alone was found to have no significant effect on the survival of *B. atrophaeus*, *B. pumilus*, or *B. subtilis* over the course the short-term exposures tested here compared to the lab controls maintained at a sea level pressure of 1013 mbar (*p* > 0.05) (Figure 3, Figure 4, Figure 5, Figure 6 and Figure 7). However, longer-term exposures to space vacuum have reported between −0.5 to −2 logs for 6 and 69 months for *B. subtilis* spores maintained as dried monolayers on metal coupons (see Figure 1 and references in Schuerger et al. [17]). All interactive assays described below were conducted under a background pressure of 0.05 (Figure 3) to 10^−6^ (Figure 4, Figure 5, Figure 6 and Figure 7) mbar maintained for 48 h. There was no evidence in the current experiments, or in the literature reviewed (see [29,30]), that the difference between 0.05 and 10^−6^ mbar had affected the results described herein. 

In contrast, in all VAC + HEAT assays (Figure 3 and Figure 7), the presence of vacuum increased the lethality of high temperature (i.e., 100 °C tested here) by as much as −6.5 logs for *B. pumilus* and approx. −4 to −5 logs for *B. atrophaeus* and *B. subtilis*. Thus, the synergism between VAC + HEAT had a significantly greater effect on the UV-resistant strain of *B. pumilus* SAFR-032 as compared to the other two *Bacillus* spp. The results are consistent with other studies in which vacuum increased the lethality of high temperatures at 59–60 °C by −0.5 logs [31,32,33] and at 100 °C by −6 logs [17]. In one interesting study, the synergistic effects of vacuum applied during long-term heating assays against the heat-tolerant *Bacillus* sp. ATCC 29669 boosted the overall lethality of high temperatures between 125 and 170 °C by several to many orders of magnitude under vacuum [34]. However, synergism appeared to abate when vacuum and lab-air samples were exposed to 200 °C [34]. And finally, incubating *B. subtilis* spores at −193 °C (80 K) increased survival significantly compared to room temperatures of 20 °C when both were under vacuum [31]. These data support the conclusion that the lethality of warmer temperatures increases under vacuum compared to similar tests under lab pressures closer to 1013 mbar.

The inactivation kinetics of *Bacillus* spp. spores exposed to VAC + HEAT simulations followed linear models as presented in Figure 3 here—or elsewhere [17,31,32,34]—suggesting that viable outliers do not persist beyond the linear extensions of the models when samples are exposed to high temperatures under vacuum. 

Synergism was also observed between VAC + low UV simulations (Figure 4; UV flux at 3.16 AU within the asteroid belt) and between VAC + high UV simulations (Figure 5; UV flux at 1.36 AU close to perihelion for Mars), exhibiting as much as −5 to −7 logs of increased lethality for all three *Bacillus* spp. at 1, 5, and 10 min of UV exposure. The results were consistent with a sizeable body of literature depicting synergism between UV irradiation and vacuum-tested against diverse *Bacillus* spp. [35,36,37,38,39,40,41]. Synergism between VAC and UV has also been reported for the non-spore-forming bacteria *Escherichia coli* [42,43] and *Deinococcus radiodurans* [43]. The levels of increased lethality were typically several orders of magnitude in these studies depending on the lengths of exposure to the combined VAC + UV conditions. 

When all three space factors were combined into one assay (Exp-4), the overall bioburden reductions reached approx. −7 logs (Figure 7) after only brief exposures to HEAT (i.e., only 1.25 h at 100 °C) and UV (i.e., only 2 min during the heat-pulse) in a VAC background of 10^−6^ mbar for 48 h. Figure 2B depicts one example of the overall timing of these three factors. Longer times for either HEAT or UV yielded 100% inactivation for all test samples during the three-factor experiments, meaning that no detectable viable spores were recovered. Exp-4 (i.e., VAC + HEAT + UV) required sublethal doses of each parameter to test for synergism. To measure synergistic interactions with predictive knowledge on how each factor has contributed to the overall biocidal effect, only experiments with sublethal dosages can be run. For example, if a 24 h concomitant VAC + HEAT + UV simulation were conducted, how could one resolve the contribution of each factor when results yielded zero survivors? 

Linear models were chosen here to represent the inactivation kinetics for combinations of VAC, HEAT, and UV exposure. In most cases, the linear models fit the data best. However, exceptions for biphasic plots for VAC + high UV are noteworthy. For spores of all three *Bacillus* spp., very steep inactivation kinetics were observed for the 1st phases in the high UV exposures up to approx. 5 min followed by a tailing effect due to single-digit surviving *outliers* at longer time-steps (Figure 6; Table S4). Schuerger et al. [26] reported similar effects of tailing when seven *Bacillus* spp. were exposed to a simulated Mars surface UVC flux, but eventually, they were able to sterilize aluminum coupons after longer exposures. Tailing is generally not observed for the heat sterilization of microbes but can be observed with UV sterilization processes due to the subtle shading effects of spores entrapped in pits, cracks, other surface defects or present as multi-layered spore aggregates [23]. 

Synergism works to greatly enhance the lethality of the space environment on spacecraft bioburdens. The results presented herein focused on the interactive effects of three space conditions including vacuum, simulated solar heating, and simulated solar UV irradiation. The results were consistent across all assays, and synergism was observed in all multi-factor experiments. In addition, these results are consistent with a large body of literature; some of which have been cited above. In addition, synergism has been described between HEAT and UV exposures for the fungus *Aspergillus nidulans* [44]. Interestingly, one study on the interactions between vacuum and ionizing radiation (IRAD) demonstrated a sort of a “positive synergistic interaction” in which spores of *Bacillus megaterium*, *B. subtilis* var. *niger*, *Clostridium sprorgenes*, and *Aspergillus niger* were found to survive better under γ-radiation when spores were exposed under high vacuum of approx. 10^−9^ mbar versus lab air [45]. Presumably, the γ-rays ionized the gas molecules in the lab air, forming O^−^, O_2_^−^, and/or NOx^−^ radicals, which contributed to the biocidal conditions within the sealed glass vessels. 

The results presented here can be used to estimate Sterility Assurance Levels (SALs) for spacecraft surfaces exposed to combinations of VAC, HEAT, and UV irradiation during interplanetary transits. The medical equipment industry’s standard approach [19,27,28] to estimating SALs is to estimate the times required to inactivate 1 × 10^6^ spores by a specific sterilization protocol and then double those exposure times (i.e., thus achieving 10^−12^ bioburden reductions; syn. here with −12 logs). Table 1 presents SAL estimates for all linear models in Figure 3, Figure 4 and Figure 5. The SALs in Table 1 were estimated by extending the linear models to −12 logs and are similar to what can be derived following the industry standard protocol outlined above. 

SAL estimates for the slowest combination of VAC + low UV (Table 1) approached 76 min of exposure for sun-facing surfaces. The fastest times to one SAL were observed for the linear plots in Figure 5B and 5C at 2.8 and 9.5 min for *B. atrophaeus* and *B. pumilus*, respectively. Overall, these inactivation kinetics are in agreement with survival rates given for the Lunar Microbial Survival (LMS) [17] and Cruise-Phase Microbial Survival (CPMS) [18] models. In the LMS, when all factors were combined into a single prediction per lunation (14.77 d of solar illumination in each 29.53 d lunation) for lunar spacecraft, as many as −2479 logs of bioburden reduction were possible. The value equates to an accumulation of 207 SALs (i.e., [−2479 logs] ÷ [−12 logs per SAL]) for external spacecraft surfaces on the Moon per lunation. Thus, single SALs are expected to occur over extremely short periods of time within the inner solar system (Table 1; [18]), and extremely high numbers of accumulated SALs can be achieved over a few weeks to months when spacecraft surfaces are exposed to vacuum, solar heating up to at least 100 °C, and solar UV irradiation.

Results suggest that the VEEGA trajectory for the Europa Clipper (EC) spacecraft will keep the EC spacecraft within the inner solar system for up to 3.5 years [5], potentially accumulating in excess of 1.7 × 10^4^ SALs on external surfaces (i.e., 207 SALs for every 14.77 d of solar illumination on the Moon in the LMS model multiplied by 3.5 years) during exposure to cis-lunar/LEO conditions or higher (i.e., closer to Venus). In addition, even after leaving the 1–2 AU environment, the EC spacecraft’s external surfaces will continue to accumulate SAL exposures throughout the mission, increasing the confidence that launched bioburdens from the Earth are unlikely to impact the mission science [18]. Bioburden reductions on internal spacecraft surfaces will depend on the thermal conduction of heat into interior spaces [17] and on the overall long-term effects of VAC on the lethality of the interplanetary environment [18]. 

Regarding the MSR mission architecture, the best environment in which vacuum, solar heating, and solar UV will impact microbial survival is during operations in LMO for the Orbital Sample (OS) holding device and the external surfaces of the ERO spacecraft. However, no quantitative model has yet been developed to precisely model the LMO, ERO, or OS environments in regard to how quickly each phase of the MSR mission will accumulate adequate SAL values for mitigating both forward and back contamination.

And finally, what is the most biologically relevant method to represent bioburden reductions on spacecraft surfaces when the datasets are dominated by numerous *non-detect* values? Retaining treatments that were 100% *non-detects* in the plots acted to artificially flatten the linear models by artificially creating plotted means from randomly generated values in the datasets. For example, there are missing treatments for most of the 48 h plots for VAC + HEAT in Figure 3. Including the *non-detects* when all replicates were below the detection limit of the MPN assays would have implied that some spores survived around −6 to −7 logs depending on the random numbers used. If a treatment series like 12, 24, and 48 h (e.g., Figure 3) is used—and all replicates in the 48 h treatments were *non-detects*—we cannot interpret the overall lethality of the VAC + HEAT exposures after 24 h. Was full lethality achieved at 26, 32, 36.7, or 48 h? 

Thus, caution must be taken to not bias the plotted data by inserting randomly generated values for *non-detects* in treatments in which 100% of all replicates have zero recovered spores. Such a process would create mean values of randomly generated data that implied some survival was present when in fact none was detected. In contrast, random numbers were used for *non-detects* in treatments in which at least one replicate had an actual detection of viable spores because now there was at least one positive detection of a few viable spores and the randomly generated values between 0 and 1 were good proxies for other *non-detects* in the data subsets. 

The analytical problem arises when using log-transformations in ANOVA to induce homogeneity of treatment means. ANOVA cannot process zero values. However, if at least one replicate in a treatment has a positive detection of spores, the other *non-detect* replicates can be represented by randomly generated numbers between 0 and 1. This issue became a dominant bias in these experiments because there were numerous *non-detects* in most of the outlying VAC + HEAT and VAC + high UV assays. 

## 5. Conclusions

Multi-factorial experiments are required to develop robust quantitative models on the lethality of the space environment and to accurately predict synergistic interactions of biocidal conditions on spacecraft bioburdens. The results here confirm earlier data (e.g., LMS [17] and CPMS [18] models) that synergism among the three biocidal conditions of vacuum, solar heating up to 100 °C, and solar UV irradiation yields extremely fast SAL exposures of a few minutes to a few hours for most external spacecraft surfaces between Venus (0.7 AU) and Mars (1.5 AU). When interplanetary spacecraft are deployed and fully extended within the inner solar system, bioburdens should be eradicated on external surfaces over very short time-steps, measured in tens of minutes to several hours, and in which very high accumulated SAL exposures will occur (e.g., >1 × 10^4^ SALs discussed above) over their multi-year residence times within the inner solar system. However, most of the data presented here are only applicable to unshielded spores on external spacecraft surfaces because (i) solar UV irradiation cannot penetrate to interior spacecraft locations and (ii) high temperatures from solar heating may not extend deeply into spacecraft structures [17,18]. Between Mars and Saturn, VAC + HEAT effects on internal spacecraft structures will dominate the lethality on interior surfaces [18]. Beyond 10 AU (i.e., just outside the orbit of Saturn), VAC-only effects on the lethality of the space environment will dominate on internal spacecraft structures [18]. These results support the conclusion that microbial bioburdens on external surfaces of interplanetary spacecraft—including the EC and MSR missions—will not survive for more than a few days after reaching LEO and beginning their interplanetary transits to Europa and Mars, respectively. 

## Figures and Tables

**Table 1:** Sterility Assurance Level (SAL; −12 logs) ^a,b^ predictions for linear models in Figure 3, Figure 4 and Figure 5.

Bacterial Species	Figure 3 Data: 100C + 0.05 mb Linear Model (h)	Figure 4 Data: VAC + Low-UV Linear Model (min)	Figure 5 Data: VAC + High-UV Linear Model (min)
*Bacillus atrophaeus*	48.0	69.9	2.84
*Bacillus pumilus*	22.8	59.7	9.45
*Bacillus subtilis*	41.4	76.3	12.94

^a^ SAL predictions are based on extrapolating the linear models in the designated figures to bioburden reductions of −12 logs using the slope values from Table S6. ^b^ Abbreviations: mb = mbar; VAC = vacuum; UV = ultraviolet irradiation; h = hour(s); min = minutes.

## Data Availability

All raw data for Figure 3, Figure 4, Figure 5, Figure 6 and Figure 7 are presented as single Excel files in the Supplementary section as Tables S2 and S5. In addition, raw data from the current study are available in a University of Florida Institutional Repository (UFIR) for A.C.S. at the link https://ufdc.ufl.edu/collections/ufir/results?page=1&q=Schuerger%2CAndrew%20 [last accessed on 9 September 2024].

## Supplementary Materials

The following supporting information can be downloaded at: https://www.mdpi.com/article/10.3390/microorganisms12101976/s1, Table S1: Environmental data for Figure 2B; Table S2: Survival data for Figure 3; Table S3: Survival data for Figure 4; Table S4: Survival data for Figure 5 and Figure 6; Table S5: Survival data for Figure 7; Table S6: Data for slopes and linear models in Figure 5.

## Abbreviations

ANOVA	analysis of variance
AU	astronomical unit
CPMS	Cruise-Phase Microbial Survival model
EC	Europa Clipper
ERO	Earth Return Orbiter
HEAT	simulated solar heating
IR	infrared radiation
LMO	low Mars orbit
LMS	Lunar Microbial Survival model
LN2	liquid nitrogen
LSM	least-squares mean separation
MSR	Mars Sample Return
OD	optical density
OS	Orbital Sample holder in the MSR architecture
PAC	Planetary Atmospheric Chamber
PP	planetary protection
SAL	sterility assurance level
SRL	Sample Return Lander
UHP N_2_	ultra-high-purity nitrogen gas
UV	simulated solar ultraviolet irradiation
UVC	(200–280 nm)
VAC	vacuum
VIS	visible light (400–700 nm)
VEEGA	Venus–Earth–Earth gravity assists for the EC spacecraft
